# Effects of the DSP-toxic dinoflagellate *Dinophysis acuta* on clearance and respiration rate of the blue mussel, *Mytilus edulis*

**DOI:** 10.1371/journal.pone.0230176

**Published:** 2020-03-09

**Authors:** Pernille Nielsen, Bernd Krock, Per Juel Hansen, Bent Vismann

**Affiliations:** 1 Marine Biological Section, Department of Biology, University of Copenhagen, Helsingør, Denmark; 2 Alfred-Wegener-Institut Helmholtz-Zentrum für Polar- und Meeresforschung, Bremerhaven, Germany; University of Connecticut, UNITED STATES

## Abstract

Diarrheic Shellfish Poisoning toxins (DST) are a severe health risk to shellfish consumers and can be a major problem for the shellfish industry. Bivalve molluscs can accumulate DST via ingestion of toxic dinoflagellates like *Dinophysis* spp., which are the most prominent producers of DST. The effects of DST-containing dinoflagellate *Dinophysis acuta* on bivalve clearance and respiration rate were investigated in the blue mussel (*Mytilus edulis*) exposed to different algal densities in a controlled laboratory study. Results showed that *M*. *edulis* exposed to *D*. *acuta* displayed a reduced clearance rate compared to *M*. *edulis* exposed to equivalent bio-volumes of the non-toxic cryptophyte *Rhodomonas salina*. Furthermore, *M*. *edulis* ceased to feed on *D*. *acuta* after 1 to 4 h, depending on *D*. *acuta* densities. The quickest response was observed at the highest densities of *D*. *acuta*. The estimated total amount of DST accumulated in the *M*. *edulis* exceeded the regulatory limit for human consumption and furthermore, intoxication of the *M*. *edulis* seemed to occur faster at high cell toxicity rather than at high cell density. However, respiration rates were, similar, irrespective of whether *M*. *edulis* were fed single diets of *R*. *salina*, *D*. *acuta* or a mixed diet of both algal species. In conclusion, the DST-containing *D*. *acuta* had a severe negative effect on the clearance of *M*. *edulis*, which can affect the conditions of the *M*. *edulis* negatively. Hence, DST may cause low quality *M*. *edulis*, due to reduced feeding when exposed to DST-containing *D*. *acuta*.

## Introduction

Diarrhetic shellfish poisoning (DSP) is a gastrointestinal illness caused by human consumption of shellfish that have accumulated Diarrhetic Shellfish Toxins (DSTs). Diarrhetic Shellfish Toxins are acquired by shellfish by ingestion of DST producing microalgae. This is a major problem for the shellfish industry in most parts of the world [1]. Presence of DST-containing mussels may, if the regulatory limit of 0.160 μg okadaic acid-equivalents g^-1^ meat is exceeded, result in long-time closure of shellfish production areas, which can lead to severe economic consequences for the shellfish industry [2].

Known DST-producers include several planktonic dinoflagellate species of the genus *Dinophysis* and a few epibenthic *Prorocentrum* species [1,3]. Pelagic *Dinophysis* spp. have been associated with DSP events in most cases. Okadaic acid (OA) together with its variants dinophysistoxins (DTX) and pectenotoxins (PTX) have all been identified in species of *Dinophysis*, and to accumulate in filter-feeding bivalves e.g. reviews by [4–6]. Mussels accumulate DST and PTX primarily in the digestive gland [7] and a number of studies have shown that DST undergo molecular transformations when ingested by bivalves. OA and DTX are esterified to a range of different fatty acid ester derivatives [6,8–10]. PTX are also esterified but can be further transformed to PTX seco acids and seco acid esters [6,10,11]. The molecular transformation into fatty acid esters and seco acids has been suggested to be part of a detoxification and depuration process [12]. However, [6] showed that depuration was achieved through excreting rather than metabolizing the toxins. The different types of DST have different mechanisms of action. In mussels, OA and DTX inhibit protein phosphatases [13], whereas to our knowledge, the effects of PTX on mussels still remain to be elucidated. However, PTX have been shown to affect the cytoskeleton in human cells and have hepatotoxic effects in mice [14] and references therein.

The effects of DST and PTX on bivalve physiology and survival are hitherto poorly understood [15]. Only a few studies on the direct effects of DST and PTX on bivalve feeding exist [16–18]. The majority of studies on bivalves and DST and PTX are focused on depuration processes [6,17,19–24] or physiological effects [25,26]. The physiological effects on bivalves of other harmful algal toxins have received far more attention [27–36]. Reduced clearance rates have been observed in *M*. *edulis* exposed for one hour to the toxic dinoflagellate *Karenia mikimotoi* (= *Gyrodinium aureolum*, > 600 cells ml^-1^) [27]. Presence of the karmitoxin producing dinoflagellate *Karlodinium armiger* has been shown to cause immediately cessation of clearance in *M*. *edulis* and to kill eggs, embryos and adult individuals of *M*. *edulis* [36]. The effects of the PST producing dinoflagellate, *Alexandrium catenella* (= *Protogonyaulax tamarensis*) on seven different bivalve species have been shown to be species dependent [28]. The responses included shell-valve closure and/or siphon retraction (*Mya arenaria*, *M*. *edulis* and *Geukensia demissa*), reduced clearance rate (*M*. *arenaria*, *G*. *desmissa*), increased clearance rate (*Ostrea edulis*), mucus production (*M*. *edulis*, *Placopecten magellanicus* and *G*. *demissa*) and no response (*Modiolus modiolus* and *Spisula solidissma*).

Further, the geographical area from which the mussel have been collected also plays a role [28]. Specimens of *M*. *edulis* from three different locations were exposed to *Alexandrium catenella* (formerly described as *P*. *tamarensis*). *Mytilus edulis* from two of the localities reacted with shell-valve closure and mucus production and mortality. *Mytilus edulis* collected from the third locality readily ingested PST containing alga and no mortalities were observed. This led [28] to suggest that the differences in response of *M*. *edulis* reflect that specimens from the locality periodically exposed to dinoflagellate blooms may have evolved mechanisms permitting them to exploit the toxic organisms as food with no ill effects [28]. The above clearly shows that the physiological effects of toxic algae on bivalves are not uniform but highly species-dependent and even varies with geographical location.

Except for the study by [6], the few studies on the effect of DST and PTX on bivalve physiology made at controlled laboratory conditions all used the epibenthic dinoflagellate *Prorocentrum lima* as DST source [16,18]. Reduced clearance rates in *M*. *edulis* and *Argopecten irradians* were found when fed *P*. *lima* at densities above 1· 10^3^ and 200 cells ml^-1^, respectively [16,18]. When fed the non-toxic algal, *Thalassiosira weissflogii*, in bio-volumes equivalent to the experiments with *P*. *lima*, *A*. *irradians* showed no reduction in clearance rate and [18] concluded *P*. *lima* to have a direct toxic effect on *A*. *irradians*. Although re-suspended *P*. *lima* in nature will be available for suspension feeders the most likely source of *in situ* DST contamination of suspension feeding bivalves are *Dinophysis* spp.

The lack of knowledge on the effects of DST and PTX-containing *Dinophysis* species on bivalve physiology is due to the earlier incapability to cultivate *Dinophysis* species. Currently, techniques for cultivation of *Dinophysis* species have been developed [37] and the physiological effects of DST and PTX -containing *Dinophysis* sp. on bivalve physiology can now be studied in detail. Clearance and respiration rates represent key variables in the physiology and growth of suspension feeding bivalves and are most likely to reflect any toxic effects caused by toxic algae [28,38].

In the present study, clearance and respiration rates of the blue mussel *M*. *edulis* were studied at different densities of the mixotrophic, DST and PTX-producing dinoflagellate, *Dinophysis acuta* and compared to equivalent bio-volumes of the non-toxic cryptophyte alga *Rhodomonas salina*. The research questions were: 1) Does *M*. *edulis* reduce the clearance rate when exposed to *D*. *acuta*? 2) If there is a reduction in clearance rate, is it caused by *D*. *acuta* toxicity or saturation of the alimentary canal? 3) Will ingestion of *D*. *acuta* increase the *M*. *edulis* respiration rate, possibly as a consequence of depuration costs?

## Materials and methods

### Sampling and maintenance of *Mytilus edulis*

Blue mussels, *M*. *edulis*, were collected in April 2012 at a mussel farm in the Limfjorden, Denmark (56°47’15.54”N; 8°54’57.12”E), and transported to the Marine Biological Laboratory, Helsingør, Denmark. The *M*. *edulis* were placed in four 13-litre maintenance tanks (50 *M*. *edulis* per tank) with flowing, fully aerated seawater from the laboratory water system (temperature 10–13°C and salinity ~30). The *M*. *edulis* were allowed to acclimate to temperature and salinity for at least three weeks prior to experiments. During this period, the *M*. *edulis* were fed three to four times a week with suspensions of *Rhodomonas salina*. At each feeding, 2–3 litres of algal suspension (10^6^ cells ml^-1^) were added to each maintenance tank and the water supply was turned off for 1–2 h. The day after feeding, the maintenance tanks were cleaned for faeces.

### Cultivation and bio-volume of algae

Four different protist cultures were used in the present study: The cryptophytes *R*. *salina* (K-0294, NORCCA) and *Teleaulax amphioxeia* (K-0434, NORCCA), a ciliate *Mesodinium rubrum* (MBL-DK2009), and the dinoflagellate *Dinophysis acuta* (DANA-2010). All protist cultures were grown in F/2 medium based on autoclaved natural seawater with a salinity of 32 ± 1 and pH 8.0 ± 0.1. All cultures were kept in a temperature-controlled room at 15 ± 1°C and exposed to an irradiance of 130 μmol photons m^-2^ s^-1^ (PAR) in a light:dark cycle of 16:8 h [39], except for *R*. *salina* that was grown under continuous light [40] and with aeration.

The cultures of *D*. *acuta* were fed twice a week with a culture of the ciliate *M*. *rubrum* (predator:prey ratio: 1:10), while the cultures of *M*. *rubrum* was fed twice a week with a culture of the cryptophyte *T*. *amphioxeia* (predator:prey ratio: 1:10). The bio-volume of *R*. *salina* was calculated as a prolate spheroid with circular cross-section ($\frac{\pi }{6}∙d^{2}∙h$, where d = diameter and h = height) and the bio-volume of *D*. *acuta* as a prolate spheroid with elliptic cross section ($\frac{\pi }{6}∙a∙b∙h$, where a = apical height, b = transapical axis and h = height) [41]. Using the above equations, the bio-volume of *R*. *salina* and *D*. *acuta* was calculated to 105 μm^3^ $\left(\frac{\pi }{6}∙5^{2}∙8\right)$ and 29.5·10^3^ μm^3^ $\left(\frac{\pi }{6}∙35∙23∙70\right)$, respectively. Accordingly, the bio-volume of one *D*. *acuta* cell was equivalent to approximately 280 *R*. *salina* cells.

### Preliminary experiments

First, we tested if *D*. *acuta* had a detrimental effect on *R*. *salina* when kept together. An experiment with six replicate 65-ml bottles containing a mixture of 28 *D*. *acuta* cells ml^-1^ and 3.4 · 10^3^ *R*. *salina* cells ml^-1^ and six replicate 65-ml bottles with only 3.4 · 10^3^ *R*. *salina* cells ml^-1^ were prepared by adding the algal cultures to autoclaved seawater (salinity 32 ± 1). The bottles were kept in a temperature-controlled room at 15 ± 1°C and mixed by inversion of the bottles every five to ten minutes. After incubation for 2 h, the algal densities were determined by cell counts (see below), and we did not observe any detrimental effect of *D*. *acuta* on *R*. *salina* (S1 Fig).

Finally, control experiments were carried out to determine the natural sedimentation of the two algal species, *R*. *salina* and *D*. *acuta*, at the different densities (Table 1). Similar experimental conditions as in the clearance rate experiments (see below) were established but with no presence of *M*. *edulis*. The first water sample was withdrawn after two minutes of mixing. Additional water samples were withdrawn every 15 min for up to an hour. The algal densities remained constant during the duration of the control experiments (1h; S2 Fig).

**Table 1:** Algae densities of *Rhodomonas salina* and *Dinophysis acuta*.

*D*. *acuta* (cells ml^-1^) / (μm^3^)	*R*. *salina* (cells ml^-1^) / (μm^3^)	Mix of *D*. *acuta* + *R*. *salina* (cells ml^-1^) / (μm^3^)
40 / 1.2 · 10^6^	11.3 · 10^3^ / 1.2 · 10^6^	28 + 3.4 · 10^3^ / (8.3 · 10^5^ + 3.6 · 10^5^) = 1.2· 10^6^
28 / 8.3 · 10^5^	8 · 10^3^ / 8.4 · 10^5^	
14 / 4.1 · 10^5^	4 · 10^3^ / 4.2 · 10^5^	

Targeted algal densities (cells ml^-1^) and bio-volume (μm^3^) of *Dinophysis acuta* and *Rhodomonas salina* in each of the experiments. Note that the algal densities of each species represent equivalent bio-volumes. The mixture of the *D*. *acuta* and *R*. *salina* is equivalent to the highest algal densities in terms of bio-volume of cells.

### Clearance rate of *Mytilus edulis* exposed to *Dinophysis acuta*

Clearance rates of the *M*. *edulis* were studied at different algal densities (Table 1) of either the non-toxic alga *R*. *salina* (4 · 10^3^, 8 · 10^3^ and 11.5 · 10^3^ cells ml^-1^), the DST and PTX-containing *D*. *acuta* (14, 28 and 40 cells ml^-1^) or a mixture of the two algal species (*D*. *acuta*: 28 cells ml^-1^ and *R*. *salina*: 3.4 · 10^3^ cells ml^-1^). The different algal densities used in the clearance rate experiments (see below) are listed in bio-volume and cell densities (Table 1). *Rhodomonas salina* densities were selected based on the study by [42], in which clearance rate of *M*. *edulis* was high and constant at *R*. *salina (= R*. *baltica)* densities of 2 · 10^3^–6 · 10^3^ cells ml^-1^ for at least eight hours, whereas at >15 · 10^3^ cells ml^-1^ a decrease in clearance with time was observed [42]. The total bio-volume of the mixed *R*. *salina* and *D*. *acuta* diet were equal to the highest bio-volume used in the single algal species experiments and had a potential equal amount of DST and PTX as the intermediate density of *D*. *acuta*.

Nine randomly selected *M*. *edulis* were selected the day prior to the start of experiments and positioned individually in the maintenance tank on nine glass plates where they embyssed. This was done to avoid stress due to breakage of byssus [43], when transferring the *M*. *edulis* from the maintenance tank to the experimental set-up. For each algal density (Table 1) two experiments were made, each including three 12-litre experimental aquariums (in total n = 6). The aquariums were placed in a temperature-controlled room (13 ± 1°C) and 4 l of seawater (salinity ~30) was added to each aquarium. The water in the aquariums was aerated and mixed by small centrifugal pumps during the entire experimental period. At the start of experiment, the appropriate volume of algal suspension was added to the aquariums and allowed two minutes to become homogeneously mixed. Subsequently, three individual *M*. *edulis* on glass plates were positioned randomly in each aquarium. Each clearance rate measurement was carried out in one h cycles repeated for up to 5 h according to the standard method used by [44]. In each aquarium, the decrease in algal density was monitored every 15–20 minutes for 60 minutes by withdrawal of water samples (25 ml). After each cell count (see below) the remaining water samples of ≥19 ml were transferred back to the respective aquarium. A new 60 minutes measure period was initiated by re-establishing the initial algal density by addition of algal suspension after removal of equal water volumes to ensure a constant volume of 4 l. In the experiments with mixture of *R*. *salina* and *D*. *acuta*, water samples were withdrawn every 40 minutes for two hours due to the extended analysis time (see below) before the initial algal density was re-established. The clearance rate experiments were terminated if either the clearance rate reached zero or the experimental period of 5 h was reached.

The density of *R*. *salina* and *D*. *acuta* in the water samples was measured in triplicates (1 ml each) using an electronic particle counter (Coulter counter, Multisizer 3) equipped with a 100 or a 280 μm aperture tube for the measurement of *R*. *salina* and *D*. *acuta*, respectively. In the mixed suspensions of *R*. *salina* and *D*. *acuta*, the density of *R*. *salina* was measured as above, whereas the *D*. *acuta* was determined by manually counting triplicates of fixed samples (acidic Lugol’s) using an Olympus CK2 inverted microscope at 40-200x and 1-ml Sedgewick-Rafter sedimentation chambers. This was due to the fact that none of the two aperture sizes could count both algal sizes at the same time.

The observed clearance rates (CR_obs_, l h^-1^ ind^-1^) were calculated using Eq 1. If *M*. *edulis* in each aquarium cleared the water from algae at a constant rate during the experiments, the decrease in algal density as a function of time will be linear in a semi-log plot (S3 Fig). Only experiments with a slope significantly different from zero and with R^2^ > 0.80 (mean R^2^ = 0.89 ± 0.05 for measurements with and R^2^ > 0.80) were used to calculate the clearance rate.

$${\mathrm{CR}}_{\mathrm{obs}}=\frac{v}{t∙n}∙\mathrm{Ln}\left(\frac{C_{0}}{C_{t}}\right)$$ (1)

Where V = water volume (l), t = time (hour), n = number of *M*. *edulis* (3) and C_0_ and C_t_ = algal densities (cells l^-1^) at time 0 and t, respectively. All CR_obs_ were corrected for the negligible sedimentation effects in the control aquariums without presence of *M*. *edulis* (S2 Fig).

The CR_obs_ were standardized to weight specific clearance rate (CR_dw_, l h^-1^ g^-1^) using Eq 2.

$${\mathrm{CR}}_{\mathrm{dw}}={\left(\frac{{\mathrm{dw}}_{s}}{{\mathrm{dw}}_{0}}\right)}^{0.66}∙{\mathrm{CR}}_{\mathrm{obs}}$$ (2)

Where dw_s_ = 1, dw_0_ = average soft tissue dry weight (g) of the three *M*. *edulis* in each aquarium, 0.66 = the allometric exponent [45] and CR_obs_ = the observed clearance rate (l h^-1^ ind^-1^).

### Ingestion rate estimation

During experiments, visual inspection of the aquariums gave no indications of pseudofeces production. In addition, after the experiments empty *D*. *acuta* thecae were found in both the stomach-content (inverted-microscopy) and in fecal material (epifluorescence microscopy), which imply that the *M*. *edulis* actually ingested and digested the DST and PTX-containing *D*. *acuta* cells during the experiments. The retention efficiency of *M*. *edulis* when clearing *R*. *salina*, *D*. *acuta* or a mixed diet of the two algae was set to 100%. Hence, the number of cells ingested was calculated by multiplying the observed clearance rate (l h^-1^ ind^-1^) with the mean algal density (cells l^-1^). The total bio-volume ingested (μm^3^) was calculated by multiplying the number of ingested cells with the cell bio-volume of either *D*. *acuta* or *R*. *salina*.

### Oxygen consumption on individual *Mytilus edulis* exposed to *Dinophysis acuta*

**Fig 1:**
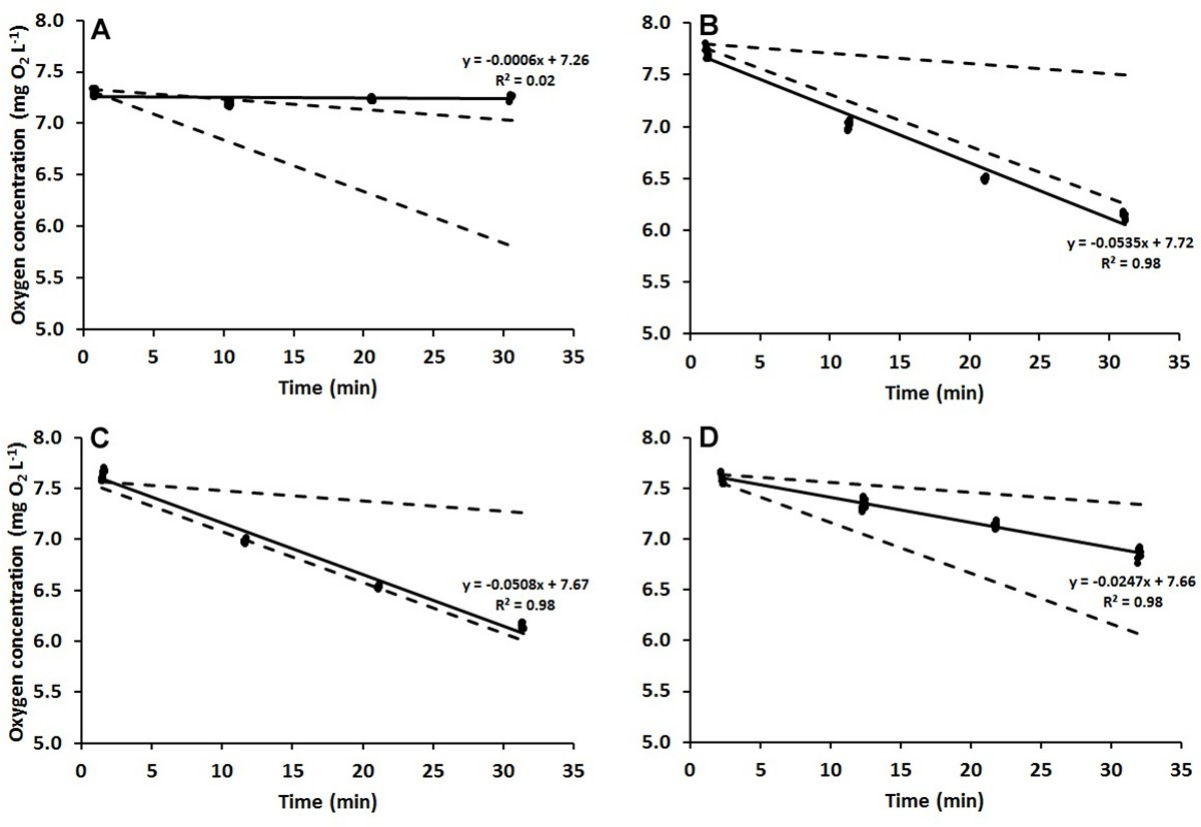
Oxygen concentration in the respiration chamber. The decrease in oxygen concentration in the respiration chambers as a function of time in four different *Mytilus edulis* oxygen consumption measurements (filled circles). A) inactive mussel (no stirring), B-D) active mussels. Linear regression lines, regression equations and R^2^ are given on the figure. The theoretical decrease in oxygen concentration in the respiration chamber (broken lines) calculated for the highest oxygen consumption (0.8 mg O_2_ h^-1^ g^-1^) from literature ([49,50] and for the lowest (0.13 mg O_2_ h^-1^ g^-1^) [48].

The oxygen consumption experiments were conducted immediately after termination of each clearance rate experiment. The nine glass plates with individual *M*. *edulis* were gently moved from the clearance rate set-up into nine individual small transparent glass respiration chambers (60 ml). Because of the small chamber volume, the *M*. *edulis* themselves supplied the stirring of the water during measurements. Whether the *M*. *edulis* could stir the water sufficiently to allow oxygen consumption to be measured reliably was tested in a pilot study. In the literature the oxygen consumption of *M*. *edulis* has been found in the range of 0.13–0.8 mg O_2_ h^-1^ g^-1^ [46–50]. The oxygen consumption rates from literature can be standardized (exponent as in Eq 2) to the weight of the *M*. *edulis* used in the present study and further expressed as the concomitant decrease in oxygen saturation in a respiration chamber with the volume of 60 ml. Four representative examples of measured decreases in oxygen concentration obtained in the pilot-study together with the range of decrease in oxygen concentration taken from the literature are presented in Fig 1. Three of the *M*. *edulis* shown (Fig 1B–1D) stirred the water sufficiently and the measured oxygen consumption rates were in full accordance with the literature. Likewise, an inactive *M*. *edulis* (Fig 1A), which did not stir the water to a sufficient degree, could readily be identified. Hence, the pilot study verified that when active, the *M*. *edulis* did stir the water sufficiently and the set-up was appropriate to measure reliable oxygen consumptions. In the present experiments, the longest lasting measurement was 40 minutes, which at the highest oxygen consumption found in literature correspond to a decrease in chamber oxygen of 2 mg O_2_. Therefore, the lowest theoretical oxygen saturation at the end of experiment can be estimated to approximately 77%, which is not considered critical for *M*. *edulis* to respire fully aerobic.

The *M*. *edulis* were left undisturbed for 20 minutes after transfer to their individual respiration chambers, before the chambers were closed with airtight lids and measurements done. After closure of the chambers, the oxygen concentration in each chamber was measured every 10 minutes for 20–40 minutes. After the final oxygen measurement, *M*. *edulis* were gently removed from the chambers and the oxygen consumption in the chambers was measured without presence of *M*. *edulis*. The oxygen concentration in the chambers was measured from the outside (minimal disturbance of the *M*. *edulis*) using a fiber-optic oxygen probe (Fibox 3, Minisensor oxygen meter, Presens Precision Sensing GmbH, Germany) where the oxygen-sensitive sensor spots were glued to the inside of the respiration chambers. To ensure reproducible readings, each chamber was equipped with a guide for precise placement of the optical fiber. Measurements where *M*. *edulis* were inactive resulted in no significant decrease in oxygen concentration (e.g. Fig 1A) and these measurements were excluded from the calculations.

The decrease in oxygen concentration in the individual chambers was plotted against time and a linear regression was made (only measurements with R^2^ > 0.80 [mean R^2^ of all measurements was 0.95 ± 0.04] were used in calculations). The weight specific oxygen consumption (MO_2_, mg O_2_ h^-1^ g^-1^) was calculated according to:

$${\mathrm{MO}}_{2}={\left(\frac{1}{\mathrm{dw}}\right)}^{0.7}∙\left(({‐\delta O}_{2}∙V)-{\mathrm{MO}}_{2_{\mathrm{blind}}}\right)$$ (3)

where dw = dry weight (g) of soft tissue, 0.7 = the allometric exponent [51], δO_2_ = the slope of decrease in oxygen concentration (mg O_2_ l^-1^ h^-1^) during measurement, V = volume of chamber (l) and ${\mathrm{MO}}_{2_{\mathrm{blind}}}=the\;oxygen\;uptake\;of\;the\;chamber\;without\;presence\;of\;a\;M.edulis.$

### Determination of shell length and dry weight of *Mytilus edulis*

At the end of the oxygen consumption measurements, the shell length of the individual *M*. *edulis* was measured with a digital caliper (± 0.1 mm). The *M*. *edulis* tissue was separated from the shells and dried at 80°C until no further weight loss was recorded (≥ 3 d). The samples were allowed to cool to room temperature in a desiccator and subsequently weighed to the nearest mg.

### Determination of DST and PTX in *Dinophysis acuta*

Three samples (0.5–2.5 ml) for toxin analysis were taken from each batch of *D*. *acuta* culture at the day of experiments, because the toxicity of *D*. *acuta* varies with time [39]. The samples were transferred to spin-filters and centrifuged at 400 g for two minutes. Filtrates were removed and the spin-filters were stored at -18°C until further analysis. The samples were analyzed for the toxin groups OA, DTX and PTX according to the procedure below.

The spin filter samples were extracted with 150 μl methanol (100%) and incubated for 1 h before they were centrifuged at 800 g for two minutes. The extract was transferred to a 2-ml glass HPLC vial with a 250 μl glass insert. Measurements were done on an SCIEX-4000 Q Trap (Sciex, Darmstadt, Germany) triple quadrupole-linear ion trap hybrid mass spectrometer equipped with a TurboSpray® interface coupled to a model 1100 LC (Agilent, Waldbronn, Germany). The LC equipment included a solvent reservoir, in-line de-gasser (G1379A), binary pump (G1311A), refrigerated auto sampler (G1329A/G1330B) and temperature-controlled column oven (G1316A). PTX were measured in the positive ionization mode as described by [52], whereas OA and DTX-1b were measured in the negative ionization mode using large volume injection (50 μl). Further details of the method are described in [39]. It should be noted that in the experiments with 14 *D*. *acuta* cells ml^-1^ all samples were re-analyzed, because the analysis showed erroneously low concentrations of OA and DTX-1b (a factor 10 lower). However, due to reduced sample volumes available after the first measurements, it was not possible to make a direct re-analysis of OA and DTX-1b. Consequently, the samples were only re-analyzed for PTX-2 and afterwards the OA and DTX-1b concentrations were estimated using the PTX-2:OA and PTX-2:DTX-1b ratio found in the first analysis and the re-analyzed PTX-2 concentration. The calculated OA and DTX-1b concentrations seemed more reliable (i.e., within the same range as the other experiments).

The total amount of each toxin group ingested by the *M*. *edulis* used in the experiments was calculated as the average toxin amount (pg cell^-1^) multiplied with the number of cells ingested (cells ind^-1^).

### Statistical analysis

Statistical analysis was made using GraphPad Prism version 7.0e for Mac (GraphPad Software, San Diego California USA, www.graphpad.com). Homogeneity of variances and normality were tested for all data sets according to Bartlett’s test, D’Agostino and Pearson omnibus normality test before further statistical analysis. The algal densities for each algal species were tested using One-way ANOVA followed by Tukey’s multiple comparison tests. The temporal development of the weight specific clearance rates for a given algal density were analyzed with repeated measures ANOVA. Repeated measures ANOVA followed by Sidak’s multiple comparisons test were used for comparison of weight specific clearance rates for experiments with the same bio-volume of the different algal species (e.g. 11.3 · 10^3^ *R*. *salina* cells ml^-1^ vs. 40 *D*. *acuta* cells ml^-1^), whereas the Mann-Whitney U-test (equal variance test failed) were used for pairwise comparison of weight specific clearance rates for each of the algal species in the mixed diet experiment. Kruskal-Wallis H test followed by Dunn's multiple comparison tests (equal variance test failed) were used to analyse the total amount of cells ingested (cells ind^-1^), the total amount of toxins ingested (μg ind^-1^) in the experiments with *D*. *acuta* and the respiration rates of *M*. *edulis* feed the two algal species and the mixed diet. Average values are given with ±1 SD or ±1 SE for weight specific clearance rate. The significance level for all tests was set at α = 0.05.

## Results

### Clearance rate of *Mytilus edulis*

The *M*. *edulis* used in the experiments had an average shell length of 32.4 ± 1.7 mm and an average tissue dry weight of 103 ± 32 mg (n = 126). The average densities of *R*. *salina* were 11.7 · 10^3^ ± 1.37 · 10^3^, 7.49 · 10^3^ ± 758 and 4.73 · 10^3^ ± 585 cells ml^-1^ and significantly different (One-Way ANOVA; F(3,266) = 990.0, P<0.0001). The average densities of *D*. *acuta* were 38 ± 7, 28 ± 6 and 16 ± 3 cells ml^-1^, respectively and all densities were significantly different (One-Way ANOVA; F(3,257) = 218.9, P<0.0001). In the experiments with the mixture of *R*. *salina* and *D*. *acuta* the average density of each algal species was 3.22 · 10^3^ ± 116 and 26 ± 4 cells ml^-1^, respectively. For simplicity, the experiments will be referred to as the algal densities shown in Table 1 in the rest of the text.

**Fig 2:**
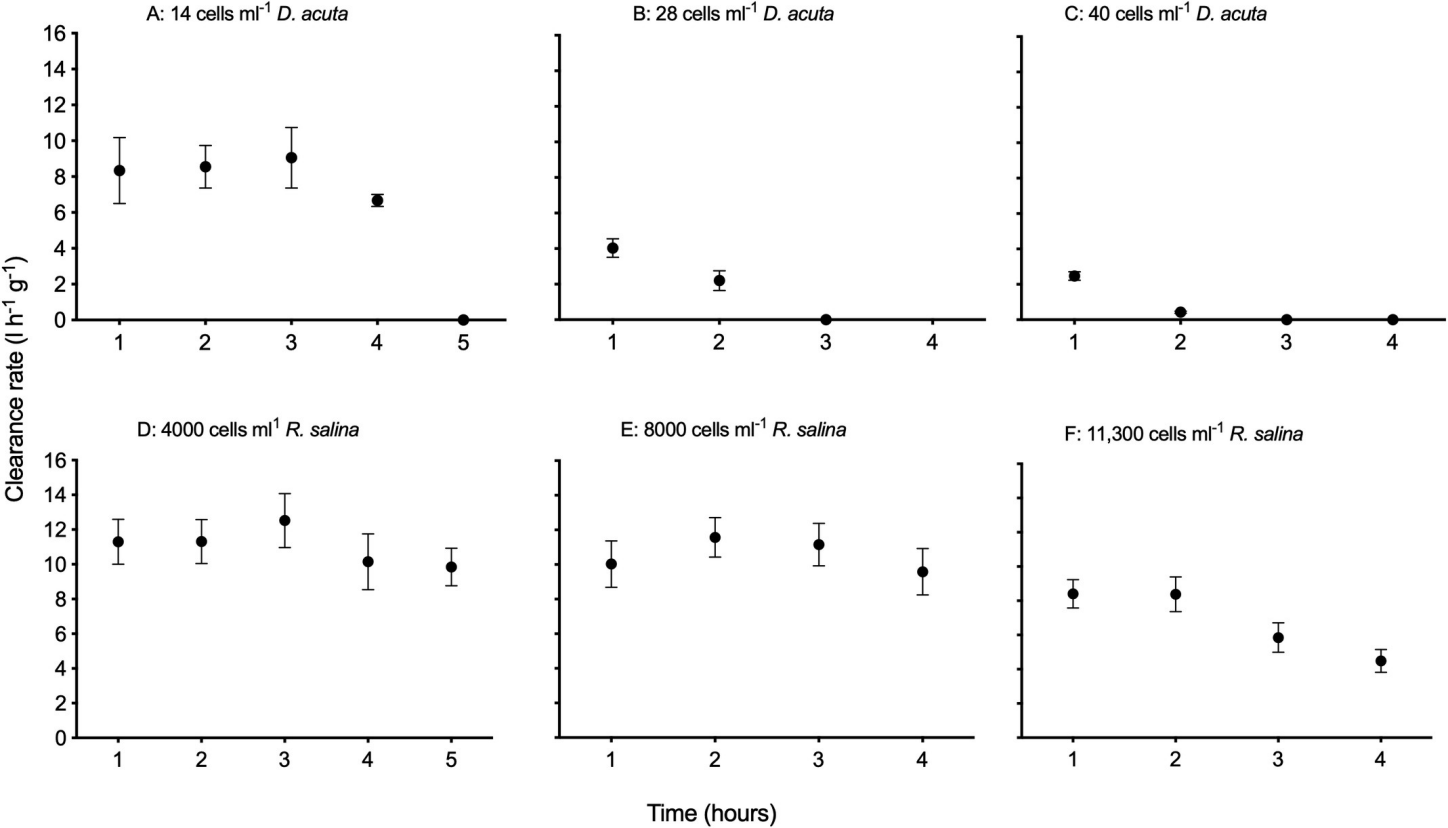
Weight specific clearance rate of *Mytilus edulis* exposed to *Dinophysis acuta* and *Rhodomonas salina*. The weight specific clearance rate (l h^-1^ g^-1^) for groups of three *Mytilus edulis* as a function of time at different algal densities of DST and PTX-containing *Dinophysis acuta* (A-C) and non-toxic *Rhodamonas salina* (D-F). Data points refer to average values and error bars to indicate ± 1 SE.

The weight specific clearance rates (CR_dw_) of *M*. *edulis* fed *D*. *acuta* of different densities (Fig 2A–2C) changed over time (Repeated Measures ANOVA; 40 cells ml^-1^ F(3,12) = 91.9, P = 0.0016), 28 cells ml^-1^ F(2,8) = 38.1, P = 0.0224 and 14 cells ml^-1^, F(3,12) = 12.3, P = 0.0070)). However, the CR_dw_ of *M*. *edulis* fed different densities of *R*. *salina* (Fig 2D–2F), only changed with time when fed 11.3 · 10^3^ cells ml^-1^ (Repeated Measures ANOVA: 11.3 · 10^3^ cells ml^-1^ F(5,15) = 27.14, P = 0.0016; 8 · 10^3^ cells ml^-1^ F(5,15) = 2.74, P = 0.1329 and 4 · 10^3^ cells ml^-1^, F(5,15) = 2.37, P = 0.0710). After the first hour of experiments (i.e., the first one hour cycle), the CR_dw_ of *M*. *edulis* had decreased by 25, 60, and 70% when exposed to 14, 28 and 40 *D*. *acuta* cells ml^-1^ as compared to *M*. *edulis* exposed to equivalent bio-volumes of non-toxic *R*. *salina* (Fig 2A–2F).

The CR_dw_ of *M*. *edulis* exposed to the two highest densities of *D*. *acuta* (Fig 2B and 2C) was during all experiments significantly different from the CR_dw_ of *M*. *edulis* fed bio-volume equivalent *R*. *salina* densities (Fig 2E and 2F) (Repeated Measures ANOVA; F(3,18) = 15.73, P<0.0001 and F(3,12) = 7.61, P<0.005, respectively). *Mytilus edulis* ceased to clear *D*. *acuta* after two and three hours when exposed to densities of 40 and 28 *D*. *acuta* cells ml^-1^, respectively. The CR_dw_ of *M*. *edulis* exposed to the lowest cell densities (14 *D*. *acuta* cells ml^-1^ and 4 · 10^3^ *R*. *salina* cells ml^-1^, Fig 2A and 2D) was not significantly different during the first four hours of the experiments (Repeated ANOVA; F(4, 24) = 6.47, P>0.05). However, after five hours the CR_dw_ of *M*. *edulis* exposed to the lowest densities of the two algal species differed significantly (Repeated ANOVA; F(4,24) = 6.47, P = 0.00013).

**Fig 3:**
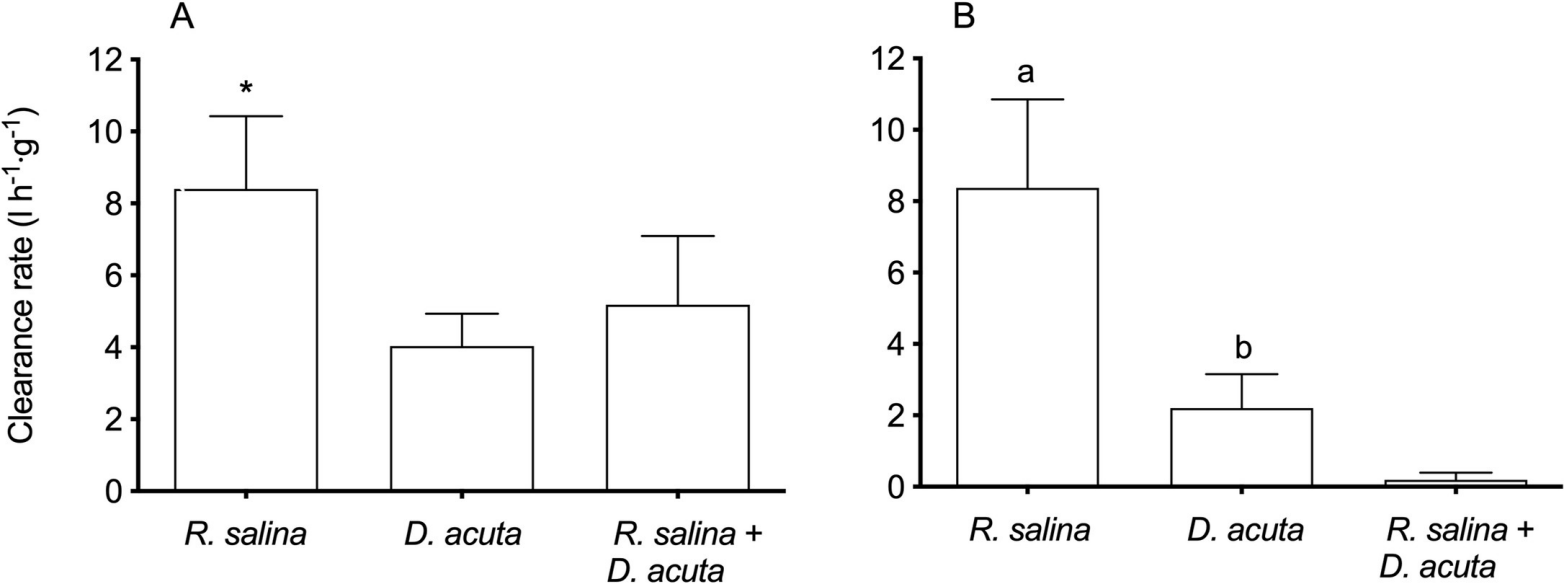
Weight specific clearance rate of *Mytilus edulis* exposed to either equivalent bio-volumes of *Rhodomonas salina* or potential equal toxin amount compared to a mixed diet. The weight specific clearance rate (l h^-1^ g^-1^) of *Mytilus edulis* within the first hour (A) and within the second hour (B) at different algal densities (cells ml^-1^) of either the non-toxic *R*. *salina* (11.3 · 10^3^ cell ml^-1^), the DST and PTX-containing *D*. *acuta* (28 cells ml^-1^) or a mixture of the two algae (*R*. *salina*: 3.4 · 10^3^ cells ml^-1^ and *D*. *acuta*: 28 cells ml^-1^). Different letters denote significant difference between treatments within the first or second hour. Bars represents average values and are shown with SD.

The average CR_dw_ of *M*. *edulis* fed 11.3 · 10^3^ *R*. *salina* cells ml^-1^, 28 *D*. *acuta* cells ml^-1^ and the mixture of *D*. *acuta* and *R*. *salina* (28 and 3.4 · 10^3^ cells ml^-1^, respectively) were within the first hour of experiment 8.4 ± 2.0, 4.0 ± 0.9 and 5.2 ± 1.9 l h^-1^ g^-1^, respectively (Fig 3A). Within the first hour, the average CR_dw_ of *M*. *edulis* fed 11.3· 10^3^ *R*. *salina* cells ml^-1^ was significantly different from the CR_dw_ of *M*. *edulis* fed either 28 *D*. *acuta* cells ml^-1^ or the mixed diet (Repeated Measures ANOVA; F(6,18) = 7.73, P<0.0001 and P = 0.0011, respectively). In contrast, no difference in CR_dw_ was observed between *M*. *edulis* fed 28 *D*. *acuta* cells ml^-1^ or the mixed diet (Repeated Measures ANOVA; F(6,18) = 7.73, P = 0.7336). Within the second hour the CR_dw_ (Fig 3B), was unchanged for *M*. *edulis* fed 11.3 · 10^3^ *R*. *salina* cells ml^-1^ (Repeated Measures ANOVA; F(6,18) = 7.73, P>0.9999). However, within the second hour the CR_dw_ for *M*. *edulis* fed 28 *D*. *acuta* cells ml^-1^ and the mixed diet were reduced to 2.2 and 0.2 l h^-1^ g^-1^, respectively (Repeated Measures ANOVA; F(6,18) = 7.73, P = 0.0243 and P<0.0001). Furthermore, *M*. *edulis* fed 28 *D*. *acuta* cells ml^-1^ had a significantly different CR_dw_ (Fig 3B) compared to the mixed diet; even though the two diets had potential the same toxin content, when fed density of 28 *D*. *acuta* cells ml^-1^ (Repeated Measures ANOVA; F(6,18) = 7.73, P = 0.046).

Within the first hour, the CR_dw_ of *M*. *edulis* fed *D*. *acuta* and *R*. *salina* in mixture was, when calculated separately for the two prey species, 5.5 ± 1.4 and 4.9 ± 2.4 l h^-1^ g^-1^, respectively, and not significantly different (Mann Whitney U-test, U = 11, N1 = 6, N2 = 6, P = 0.3095) (Fig 3A). Therefore, the two prey species were retained by *M*. *edulis* with the same efficiency when fed in mixture. Within the second hour the CR_dw_ of both prey species offered in mixture were close to zero (< 0.07 l h^-1^ g^-1^) and were not significantly different (Mann Whitney U-test, U = 13, N1 = 6, N2 = 6, P = 0.4848) (Fig 3B).

The average observed clearance rates (l h^-1^ ind^-1^) for all one h cycles in each experiment showed clearance rates of *R*. *salina* in the range of 1.8 ± 0.4–2.4 ± 0.2 l h^-1^ ind^-1^ and for *D*. *acuta* in the range of 0.7 ± 0.1–1.9 ± 0.6 l h^-1^ ind^-1^ (Table 2). In general, the clearance rates decreased with increasing *D*. *acuta* density. In the mixed diet experiment, the observed average clearance rate (calculated as the average of both species) was 1.3 ± 0.6 l h^-1^ ind^-1^ (Table 2).

**Table 2:** Measured and calculated variables in the different clearance rate experiments conducted on *Mytilus edulis* fed different algal species and densities.

Algal species	Density (cells m^-1^)	n	CR (l h^-1^ ind^-1^)	I_tot-cells_ (x10^5^ cells)	I_tot-vol_ (mm^3^ ind^-1^)	OA (pg cell^-1^)	DTX-1b (pg cell^-1^)	PTX-2 (pg cell^-1^)
*R*. *salina*	11.3 · 10^3^	6	1.8 ± 0.4	429 ± 123	4.5 ± 0.8			
	8 · 10^3^	6	2.1 ± 0.2	343 ± 96	3.6 ± 0.7			
	4 · 10^3^	6	2.4 ± 0.2	253 ± 73	2.7 ± 0.9			
*D*. *acuta*	40	4	0.7 ± 0.1	0.6 ± 0.06	1.9 ± 0.08	5.5 ± 2.5	6.6 ± 3.0	109 ± 46
	28	3	1.0 ± 0.2	0.4 ± 0.1	1.0 ± 0.1	1.7 ± 0.6	2.1 ± 0.7	73 ± 8
	14	4	1.7 ± 0.2	0.7 ± 0.2	2.2 ± 0.7	1.4 ± 0.6	1.7 ± 0.7	37 ± 16
*D*. *acuta* +	28	6	1.4 ± 0.3	0.3 ± 0.01	0.9 ± 0.3	5.1 ± 1.2	5.5 ± 1.4	29 ± 4
*R*. *salina*	3.4 · 10^3^	6	1.2 ± 0.5	38 ± 14	0.5 ± 0.1			

The algal species and initial algal density (cells ml^-1^), number of replicates (n), the clearance rate (CR, l h^-1^ ind^-1^), the number of cells ingested (I_tot-cells_, x10^5^ cells) and the total bio-volume ingested (I_tot-vol_, mm^3^ ind^-1^). In addition, the toxin content (pg cell^-1^) of okadaic acid (OA), dinophysistoxin (DTX-1b) and pectenotoxins (PTX-2) in the *D*. *acuta* experiments are given. All average values presented with SD.

### Total cell-volume ingested by *Mytilus edulis*

The total bio-volumes ingested in the experiments with different densities of *R*. *salina* and *D*. *acuta* were in the range of 2.7 ± 0.9 to 4.5 ± 0.8 and 1.0 ± 0.1 to 2.2 ± 0.7 mm^3^, respectively (Table 2). A comparison of the experiments in which *M*. *edulis* was fed the three diets with the same bio-volume (i.e., 1.2 · 10^−6^ μm^3^) showed that the total bio-volume ingested (Table 2) by *M*. *edulis* fed 11.3 · 10^3^ *R*. *salina* cells ml^-1^ was significantly different from the bio-volume ingested when fed 40 *D*. *acuta* cells ml^-1^ and the mixed diet (Kruskal-Wallis, followed by Dunn’s multiple comparison test, H = 9.35, df = 3, P = 0.0067). A comparison of the two experiments in which *M*. *edulis* were fed diets with the same cell density of *D*. *acuta*, and thereby potentially equal toxin amount (i.e., 28 *D*. *acuta* ml^-1^ and the mixed diet), revealed no difference in ingested bio-volume (Kruskal-Wallis, followed by Dunn’s multiple comparison test, H = 9.35, df = 3, P = 0.23).

The accumulated number of *D*. *acuta* cells ingested at the time *M*. *edulis* stopped clearing, was independent of the *D*. *acuta* density regardless whether offered as a single prey or as a mixed diet (Table 2, Kruskal-Wallis, followed by Dunn’s multiple comparison test, H = 9.25, df = 4, P > 0.05). On average, *M*. *edulis* stopped clearing when 50.1· 10^3^ ± 6.94 · 10^3^ *D*. *acuta* cells had been ingested, which corresponded to a bio-volume of 1.5 ± 0.2 mm^3^.

### Toxin measurements and toxin ingestion by *Mytilus edulis*

**Fig 4:**
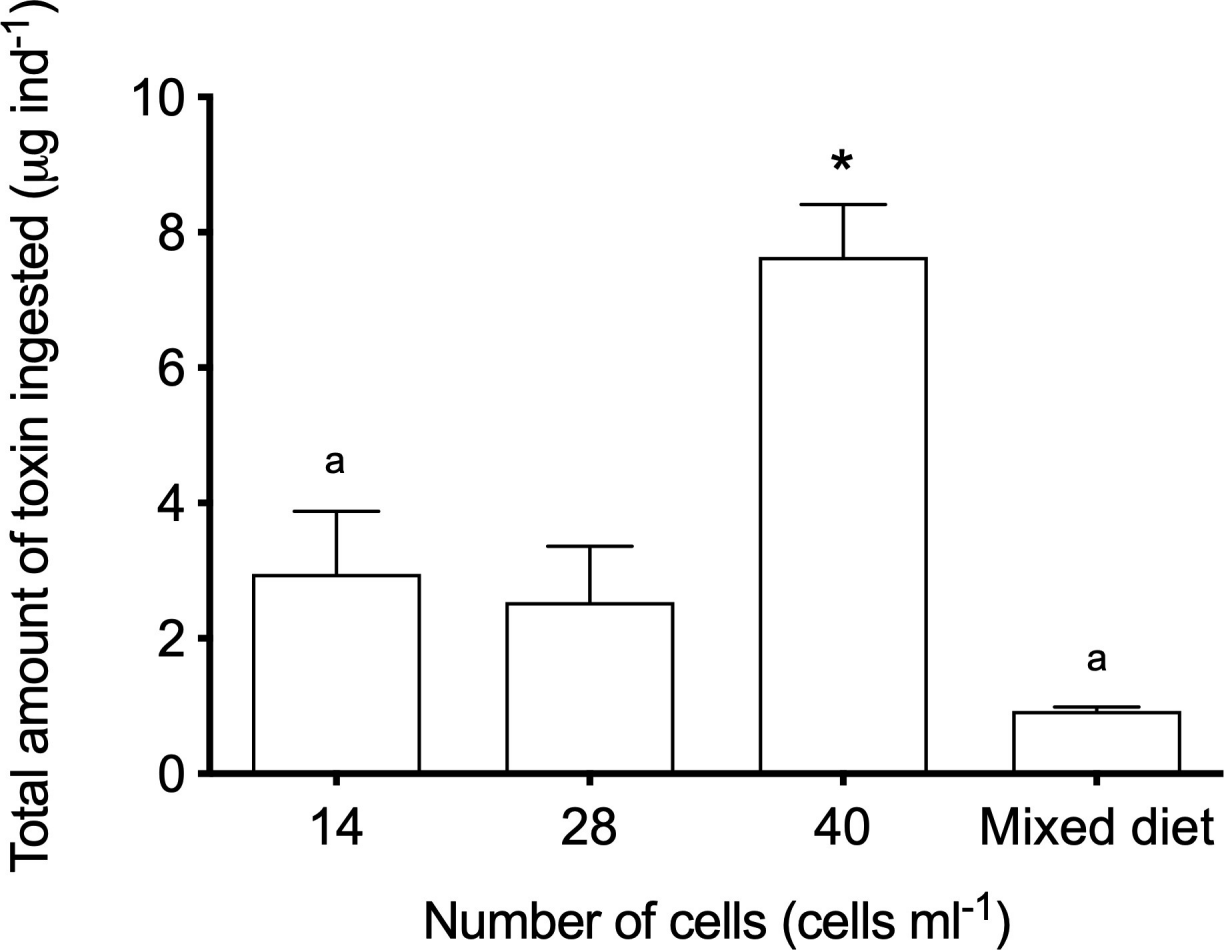
Toxin ingestion of *Mytilus edulis* exposed to *Dinophysis acuta*. The estimated total amount of OA+DTX-1b+PTX-2 ingested by *Mytilus edulis* exposed to three different densities of the DST and PTX-containing *D*. *acuta* (14, 28 and 40 cells ml^-1^) and a mixed diet (*R*. *salina*: 3.4 · 10^3^ cells ml^-1^ and *D*. *acuta* 28 cells ml^-1^). * indicates significant difference from all other treatments (P < 0.01) and ^a^ indicates significant differences (P = 0.035). Bars represent average values and are shown with SD.

The DST and PTX-2 content (pg cell^-1^) of the different batches of *D*. *acuta* varied between experiments (Table 2). The experiments with density of 40 *D*. *acuta* cells ml^-1^ had the highest content of all three toxins (OA, DTX-1b and PTX-2). Equal amounts of OA and DTX-1b were observed in the experiment with 40 *D*. *acuta* cells ml^-1^ and the mixed diet. The content of both OA and DTX-1b was lower in experiments with 14 and 28 *D*. *acuta* cells ml^-1^ compared to 40 *D*. *acuta* cells ml^-1^ (Table 2). The total amount of OA+DTX-1b+PTX-2 ingested by *M*. *edulis* (Fig 4) was significantly higher in the 40 *D*. *acuta* cells ml^-1^ experiment with the highest toxin content (Table 2) as compared to the other experiments (Kruskal-Wallis H test, followed by Dunn’s multiple comparison test, H = 10.07, df = 3, P = 0.006). Thus, the highest toxin ingestion coincided with the most pronounced clearance rate reduction and the quickest cessation of clearance activity (Fig 2C).

### Respiration rate of *Mytilus edulis*

**Fig 5:**
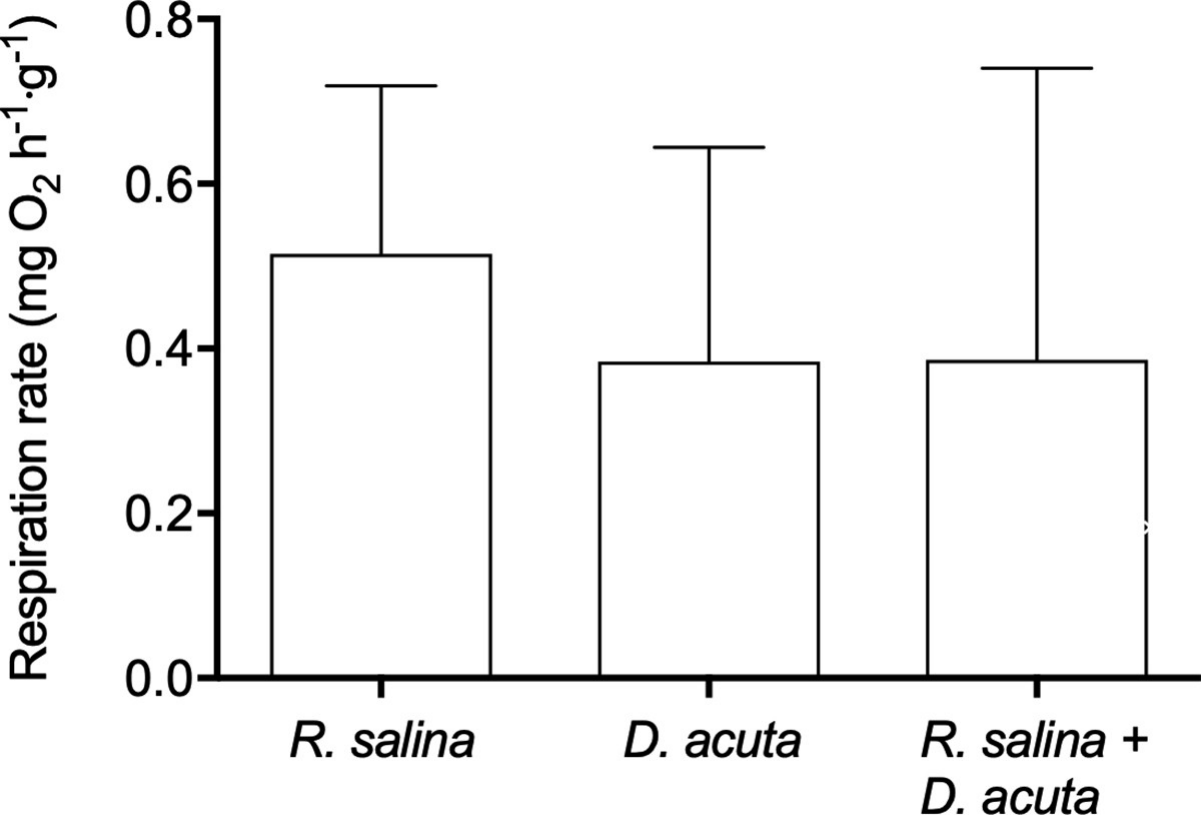
Respiration rate of *Mytilus edulis* exposed to *Rhodomonas salina* or *Dinophysis acuta*. The respiration rate of *Mytilus edulis* exposed to either non-toxic *Rhodomonas salina* (11.3 · 10^3^ cell ml^-1^), DST-containing *Dinophysis acuta* (28 cells ml^-1^) or a mixture of the two algae (*R*. *salina*: 3.4 · 10^3^ cells ml^-1^ and *D*. *acuta*: 28 cells ml^-1^). The *R*. *salina* and the mixture of *R*. *salina* and *D*. *acuta* represent equal bio-volumes, whereas the *D*. *acuta* and the mixture of *D*. acuta + *R*. *salina* represent equal toxin amounts (both 28 *D*. *acuta* ml^-1^). Bars represents average values and are shown with SD.

The average respiration rates of *M*. *edulis* when fed *R*. *salina* (11.3 · 10^3^ cells ml^-1^), *D*. *acuta* (28 cells ml^-1^) or the mixture of *D*. *acuta* and *R*. *salina* (28 + 3.4 · 10^3^ cells ml^-1^, respectively) were not significantly different (Fig 5) (Kruskal-Wallis test followed by Dunn's Multiple Comparison Test, H = 4.733, df = 2, P = 0.094). The overall average respiration rate of the three experiments was 0.42 ± 0.15 mg O_2_ h^-1^ g^-1^. At the end of measurement, the oxygen saturation in the respiration chambers had decreased to 83.0 ± 4.7%, which for *M*. *edulis* poses no problem for aerobic respiration.

## Discussion

### Effects of *Dinophysis acuta* on *Mytilus edulis* clearance

Presence of the dinoflagellate *D*. *acuta*, which produces OA, DTX and PTX [39], affected the feeding behaviour of the blue mussel, *M*. *edulis*. Clearance rates on *D*. *acuta* were lower than on the control prey *R*. *salina* (Fig 2). Furthermore, we observed *M*. *edulis* to cease feeding on *D*. *acuta* earlier with increasing *D*. *acuta* densities (Fig 2A–2C). These results indicate *M*. *edulis* to be physiologically affected by the ingestion of DST and PTX containing *D*. *acuta*.

The present study supports an earlier study that found the daily increase of OA in *M*. *edulis* is lower than what can be estimated from the theoretical clearance rate capacity of *M*. *edulis* and the DST-cell quotas [19]. The authors suggested that different mechanisms could explain the reduced rates (incl. reduced clearance, shell-valve closure, impeded absorptive capacity and increased depuration), which all may contribute to the lower levels seen in *M*. *edulis*. Furthermore, the authors also proposed shell-valve closure and reduced clearance rate to be more pronounced when *M*. *edulis* encounter higher amounts of okadaic acid. This observation was supported by our study, where the fastest reduction in clearance rate was found in the experiment with the highest available OA+DTX-1b content. In addition, when exposed to the same *D*. *acuta* density but with different cell toxicity (i.e., 28 *D*. *acuta* cells ml^-1^ vs. the mixed diet), we found significantly different clearance rates within two hours of feeding and the lowest clearance rate was observed for the mixed diet (Fig 3B), which had the highest OA+DTX-1b content compared to 28 *D*. *acuta* cells ml^-1^ (Table 2).

### Effects of cell-volume and toxin ingested on *Mytilus edulis* clearance

Can we be sure that the reduced clearance rates observed are due to DST and/or PTX? Reduced clearance rates have previously been shown for *M*. *edulis* exposed to high algal densities (e.g. [53–57]). The reduced filtration rates at high algal densities can be due to saturation of the alimentary canal (i.e., “saturation reduction” [44]). Therefore, the reduced clearance rates observed in *M*. *edulis* exposed to *D*. *acuta* observed in the present study may be caused by saturation reduction. The gut capacity of *M*. *edulis* of the size used in the present study was 4–5 mm^3^ [42]. When exposed to the non-toxic *R*. *salina* we calculated the total ingested algal volume at 4 · 10^3^, 8 · 10^3^ and 11.3 · 10^3^ cells ml^-1^ to be 2.7, 3.6 and 4.5 mm^3^, respectively. In terms of clearance rate, we only observed a reduction in *M*. *edulis* after four hours of exposure to the highest *R*. *salina* density, which is in accordance with both the gut capacity and the concept of saturation reduction [42,57]. However, when exposed to *D*. *acuta* a reduction in *M*. *edulis* clearance rates were observed at all *D*. *acuta* densities as compared to the equivalent bio-volume of *R*. *salina*. In all exposures to *D*. *acuta*, *M*. *edulis* stopped feeding on *D*. *acuta* within 3–5 hours and the total ingested algal volume was on an average calculated to 1.5 ± 0.2 mm^3^, which is below half of the gut capacity. In addition, *M*. *edulis* ingested a total volume of cells of 1.4 mm^3^ (Table 2) when exposed to the mixed diet, which also was far below the gut capacity.

The observations of empty *D*. *acuta* thecae in both the stomach-content and faeces and the absence of pseudofeces-production imply that the *M*. *edulis* actually ingested and digested the DST and PTX-containing *D*. *acuta* cells during the experiments. Digestion of *D*. *acuta* has also been observed in *M*. *galloprovencialis*, which seems to have a preference for *Dinophysis* spp. and to digest them by opening the theca of the cells [58]. In the present study, *M*. *edulis* did not display such preferential selection for *D*. *acuta* when offered in mixture with *R*. *salina*. In conclusion, *M*. *edulis* exposed to *D*. *acuta* showed reduced clearance rates, which seems most likely to be caused by the DST and/or PTX rather than saturation reduction.

Another factor, that might affect *M*. *edulis* feeding on DST and PTX-containing *D*. *acuta*, could be extracellular toxins dissolved in the seawater, since released toxins from microalgae have been shown to affect the feeding of bivalves (e.g., [59]). For DST and PTX, all three toxins (OA, DTX-1b and PTX-2) are released to the surrounding seawater [39], and thus, potentially could have caused the cessation of filtration. However, is has previously been shown that in the absence of phytoplankton but with presence of OA in the water, even at high concentration, did not induce toxicity in *M*. *edulis* [60]. Therefore, the observed effects on clearance rate seen in the present study was most likely caused by the ingestion of toxic *D*. *acuta*, and not from DST and PTX released to the water.

### Total amount of toxin ingested by *Mytilus edulis* and implications for food safety

Although toxin cell quotas in *Dinophysis* spp. are known to be highly variable, the toxin cell quotas (Table 2) of *D*. *acuta* used in the present study were within the range of toxin cell quotas measured in *D*. *acuta* cells collected in the field (e.g., [61–63]). Combined with the *D*. *acuta* densities (cells ml^-1^) used in the present study, the amount of DST and PTX in the experiments resembled observations of DST and PTX found in the field [62–67]. However, laboratory experiments with unialgal cultures can in general be questionable because they do not resemble natural conditions. In nature, *Dinophysis* blooms are rarely occurring in the absence of other non-toxic phytoplankton species. In other words, most *Dinophysis* blooms usually represent only a small proportion of the total phytoplankton community [5,68]. In spite of this, *Dinophysis* spp. even at low densities (< 10 cells ml^-1^) have been shown to result in mussels exceeding the regulatory level of 0.160 μg OA equivalent (OA + DTX + PTX) g^−1^ of shellfish meat (e.g. [5] and references therein).

No evidence of adverse acute or chronic health effects of PTX has been shown on humans [69], and as a consequence some countries do not include PTX in the OA equivalents [70]. Thus, regions (e.g., the EU) that include PTX in their calculations of OA equivalents will suffer from much longer harvesting bans and competition with production areas, where PTX have been excluded from the calculation of OA equivalent [5]. Excluding the PTX, we estimated the total accumulated amount of OA equivalents (OA+DTX-1b) to be 0.31, 0.18, 1.42 and 0.39 μg OA-eq. g^-1^ meat, when *M*. *edulis* were exposed to 14, 28, 40 *D*. *acuta* cells ml^-1^ and the mixed diet, respectively. These calculations were based on clearance rates, wet-weight of soft parts and *D*. *acuta* cell quotas determined in this study. Furthermore, in the calculation we assumed that i) the toxin accumulation efficiencies were 66 and 71% for OA and DTX-1b, respectively [6], ii) DTX-1b is as toxic as DTX1 and iii) no depuration occurred during the experiments. In conclusion, *M*. *edulis* in the present study accumulated toxins to above the regulatory limit within a few hours of feeding on DST-containing *D*. *acuta* and within a wide range of toxin cell quotas and cell densities. It has been shown that cells with high toxin cell quotas at low cell densities may lead to the same accumulation of DST in mussels as ingestion of cells with low toxin cell quotas at high cell densities [63]. Therefore, the authors concluded that the cell density multiplied with the associated toxin cell quota is decisive for the DST content of mussels [63]. Accordingly, *M*. *edulis* exposed to 28 *D*. *acuta* cells ml^-1^ and the mixed diet (28 *D*. *acuta* + 3.4 · 10^3^ *R*. *salina* cells ml^-1^) in the present study lead to similar amounts of OA+DTX-1b ingested (Fig 4), even though the toxin cell quotas of *D*. *acuta* in the two exposures were different (Table 2). The above indicates that a threshold level for OA+DTX-1b exist because *M*. *edulis* had ingested the same total amount of toxin during the incubations (Fig 3).

### Effects of *Dinophysis acuta* on *Mytilus edulis* respiration

In the present study, the observed effects on clearance rate of *M*. *edulis* exposed to *D*. *acuta* did not affect the rate of oxygen consumption (Fig 5). Similar observations have been made in previous experiments with five different juvenile bivalves (*M*. *edulis*, *Mya arenaria*, *Geukensia demissa*, *Placopecten magellanicus and Crassostrea virginnica*) that have been exposed for one hour to the PST producer *Alexandrium catenella* (reported as *A*. *tamarense*) [71]. Likewise, no difference in oxygen consumption in two bivalve species (*Ruditapes philippinarum* and *Perna viridis*) have been observed when exposed to *A*. *catenella* for six days [38]. However, the green shell mussel *Perna canaliculus* has been shown to increase oxygen consumption after one hour of exposure to *A*. *catenella* [72] and [29] observed variable respiration rates in several bivalves species when exposed to toxic *A*. *catenella* (= *Gonyaulax tamarensis*) for five days. Thus, it cannot be excluded that *D*. *acuta* could potentially influence the metabolism of *M*. *edulis* if exposed for a longer period and further studies are required on the topic.

## Conclusion

In conclusion, we have shown the clearance rate of *M*. *edulis* was reduced when fed the DST-containing (OA and DTX) *D*. *acuta* as compared to when fed the non-toxic *R*. *salina*. In addition, *M*. *edulis* ceased active feeding within a few hours at all examined densities of *D*. *acuta*. We argue that this effect was not caused by a saturation of the alimentary canal or extracellular DST dissolved in the seawater, but rather caused by a direct toxic effect of DST in cells that were ingested. Short-term exposure to DST-containing *D*. *acuta* did not have an effect on the respiration rate. However, reduced clearance or other changes associated with toxin accumulation (respiration, digestion and excretion) may affect the energy balance, which can affect the conditions of the mussels negatively.

## Supporting information

S1 Fig Verification of no detrimental effect of *Dinophysis acuta* on *Rhodomona salina*. Preliminary experiments to verify that *D*. *acuta* (28 *D*. *acuta* cells ml^-1^) in mixture with *R*. *salina* (3.4 · 10^3^ *R*. *salina* cells ml^-1^) had no detrimental effect on the latter.
(TIF)

S2 Fig Control experiments without *Mytilus edulis* present. All algae densities of either *Rhodomonas salina* or *Dinophysis acuta* remained constant during the duration of the control experiments.
(TIF)

S3 Fig Examples of semi-ln plot of clearance experiments with *Mytilus edulis* using 4·10^3^ cells ml^-1^ of *Rhodomonas salina*. New algae suspensions were added five times to re-establish initial algal density.
(TIF)
