# Supplementary figures and images for: Effects of the DSP-toxic dinoflagellate Dinophysis acuta on clearance and respiration rate of the blue mussel, Mytilus edulis
Source: PLoS One. 2020 Mar 9;15(3):e0230176. doi: 10.1371/journal.pone.0230176 (PMC7062251; doi:10.1371/journal.pone.0230176)

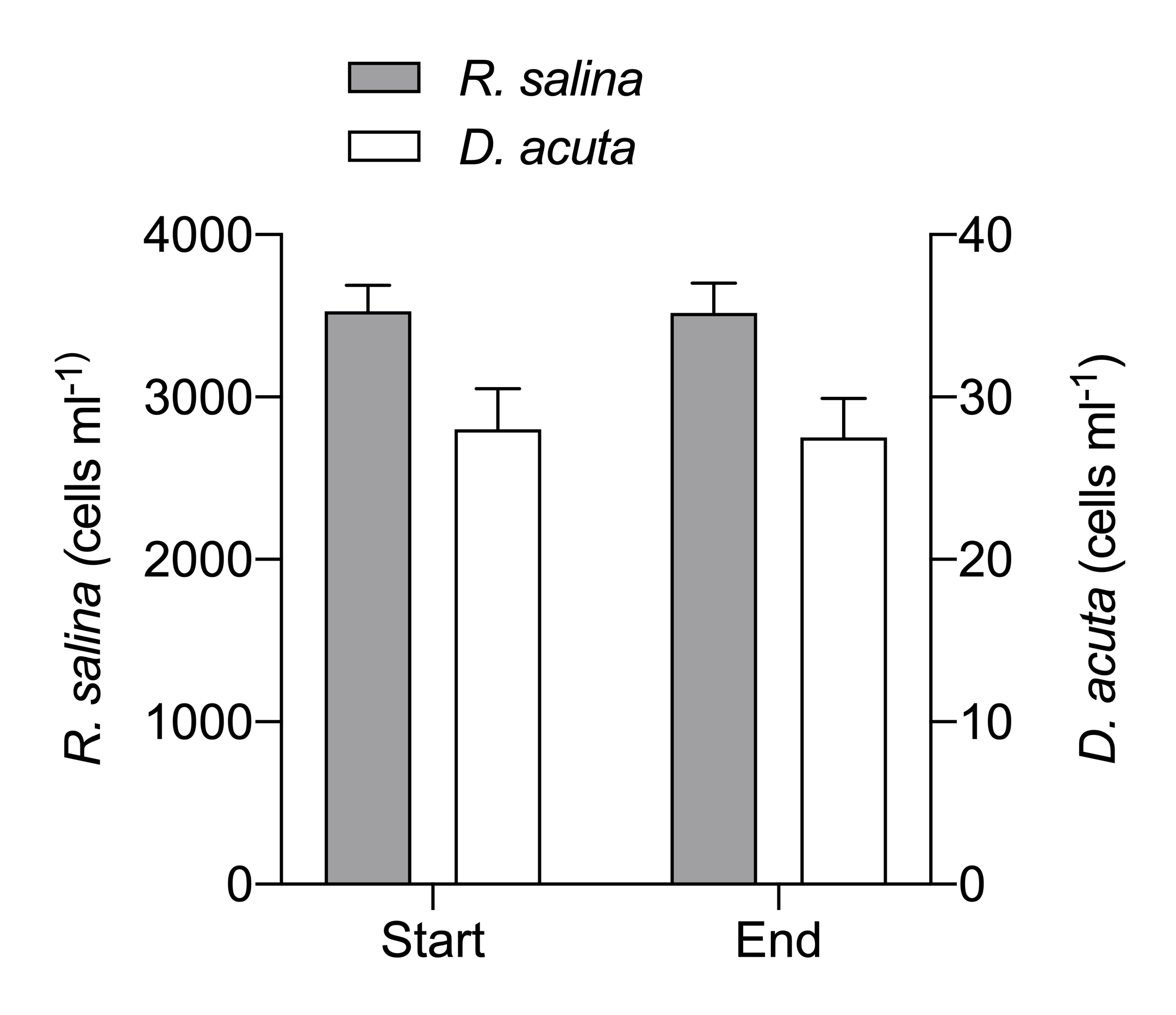

Supplement: S1 Fig — Preliminary experiments to verify that D. acuta (28 D. acuta cells ml-1) in mixture with R. salina (3.4 · 103 R. salina cells ml-1) had no detrimental effect on the latter. (TIF) [file pone.0230176.s001.tif]

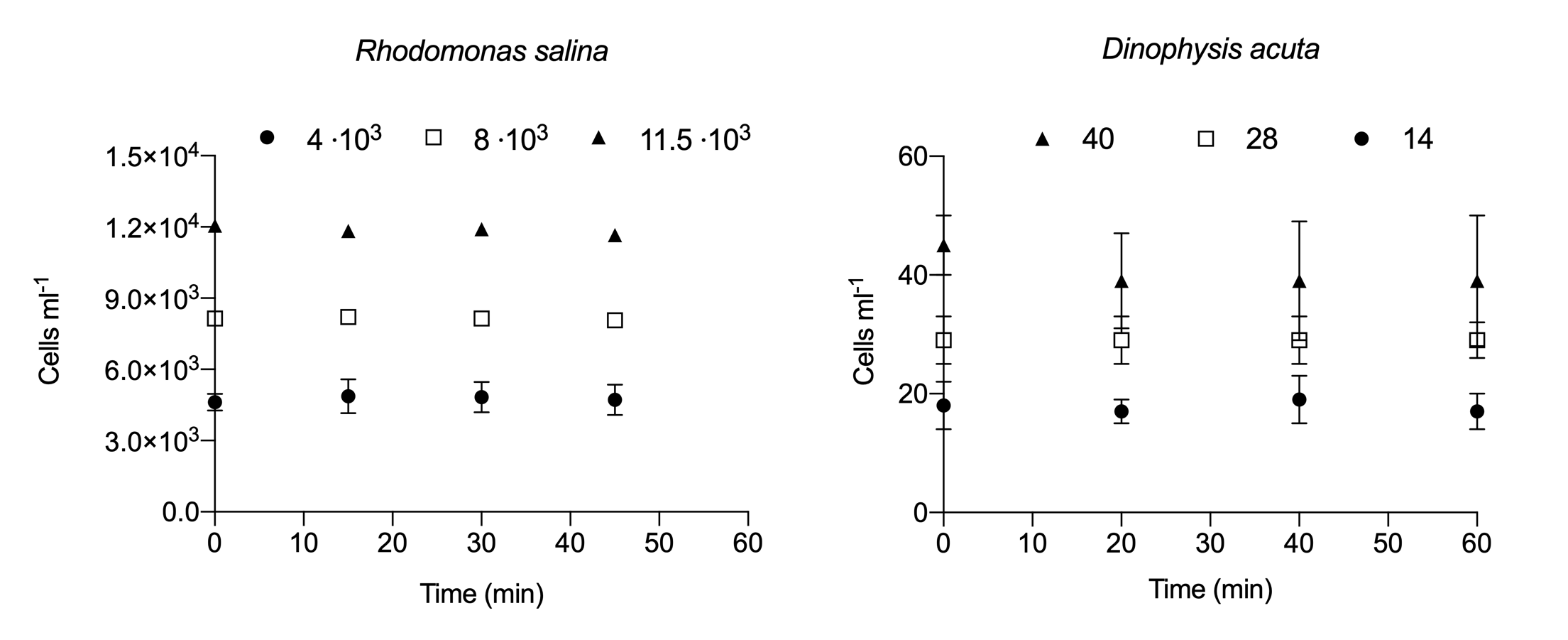

Supplement: S2 Fig — All algae densities of either Rhodomonas salina or Dinophysis acuta remained constant during the duration of the control experiments. (TIF) [file pone.0230176.s002.tif]

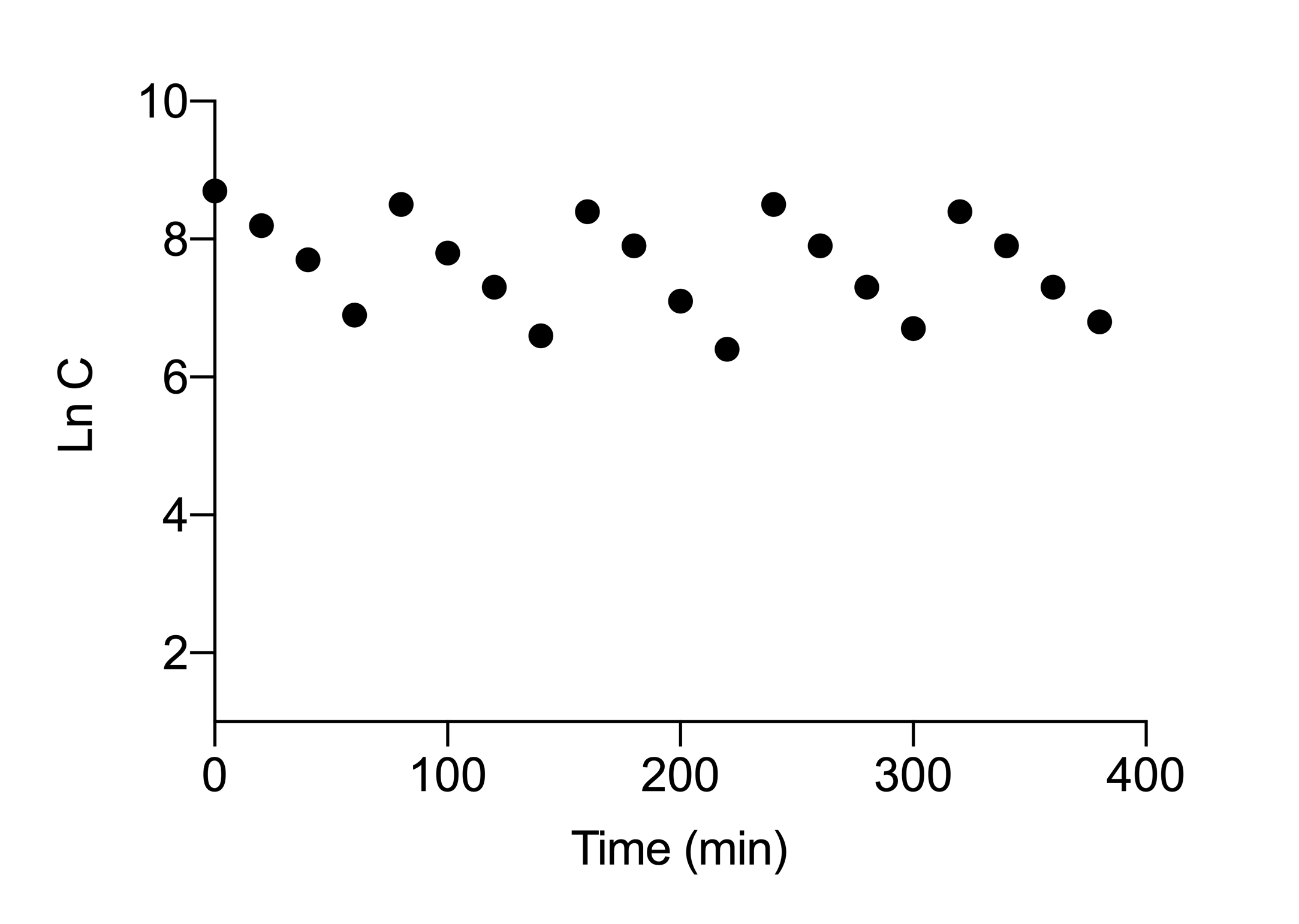

Supplement: S3 Fig — New algae suspensions were added five times to re-establish initial algal density. (TIF) [file pone.0230176.s003.tif]
